# Attractive Toxic Sugar Bait (ATSB) For Control of Mosquitoes and Its Impact on Non-Target Organisms: A Review

**DOI:** 10.3390/ijerph14040398

**Published:** 2017-04-10

**Authors:** Jodi M. Fiorenzano, Philip G. Koehler, Rui-De Xue

**Affiliations:** 1 Department of Entomology and Nematology, University of Florida, 1881 Natural Area Drive Gainesville, FL 32608, USA; pgk@ufl.edu; 2 Anastasia Mosquito Control District, 120 EOC Drive, St. Augustine, FL 32092, USA; xueamcd@gmail.com

**Keywords:** attractive toxic sugar baits, mosquito control, sugar feeding

## Abstract

Mosquito abatement programs contend with mosquito-borne diseases, insecticidal resistance, and environmental impacts to non-target organisms. However, chemical resources are limited to a few chemical classes with similar modes of action, which has led to insecticide resistance in mosquito populations. To develop a new tool for mosquito abatement programs that control mosquitoes while combating the issues of insecticidal resistance, and has low impacts of non-target organisms, novel methods of mosquito control, such as attractive toxic sugar baits (ATSBs), are being developed. Whereas insect baiting to dissuade a behavior, or induce mortality, is not a novel concept, as it was first introduced in writings from 77 AD, mosquito baiting through toxic sugar baits (TSBs) had been quickly developing over the last 60 years. This review addresses the current body of research of ATSB by providing an overview of active ingredients (toxins) include in TSBs, attractants combined in ATSB, lethal effects on mosquito adults and larvae, impact on non-target insects, and prospects for the use of ATSB.

## 1. Perspectives and Overview

The advent of synthetic insecticides occurred in the 1940s and revolutionized the way that vector control was conducted [1]. Throughout the 1950s and 1960s large-scale insecticidal treatments diminished many of the vector-borne diseases [1]. Vector control programs began to lapse with the global abatement of many vector-borne diseases in the 1960s [1]. Increased international travel and commerce over the last few decades have created more pathways for vectors and their associated diseases to spread to new places.

To control vector mosquitoes and mosquito-borne diseases, current mosquito abatement programs utilize multiple control methods that exploit mosquitoes’ known vulnerabilities while being mindful of the environment [2]. Mosquito abatement programs contend with mosquito-borne diseases, insecticidal resistance, and environmental impacts to non-target organisms. However, chemical resources are limited to a few chemical classes with similar modes of action, which has led to insecticide resistance in mosquito populations [3,4]. The World Health Organization (WHO) has urged vector control programs to discover and implement new strategies for integrated mosquito management (IMM) that are environmentally friendly, sustainable, and cost effective methods that employ insecticides with new chemical classes and modes of action [4].

To address the current mosquito control issues, novel methods of mosquito control that fulfill the WHO stipulations, such as mosquito baiting, are being explored. Mosquito baiting methods are shaped off the biological requirements of mosquitoes, mosquito ecology, and mosquito behaviors. Insect baiting to dissuade a behavior, or induce mortality, is not a novel concept.

The concept of insect baiting is first described in 77 AD by Pliny, in Historia Naturalis [5]. Pliny describes hanging a fish on a tree adjacent to foliage to lure ants away [5]. Pliny describes using a protein-based lure; however, some insects require carbohydrates in the form of plant-based sugars [6]. This requirement has led to the usage of sugars to collect and observe moths and honey bees, and kill insects by integrating a toxicant into the sugar [5]. Around 1885 attractants started to be used in baits to lure pestiferous insects away from crops to poisoned baits [5]. The benefit of plant-based attractants was discovered in 1910, with a correlation between the attractions of cabbage butterfly larva to mustard plants [5]. The concept of attractive baits took off and by 1925 hundreds of attractant, aromatic compounds were evaluated to lure orchard insects away from their natural food source [5]. Further bait studies demonstrated that an attractant is not enough to induce feeding, and a stimulant may be required to ensure consumption of baits [5]. These evaluations have led to new approaches of insect control through attractive baiting. This review addresses the current body of research of ATSB by providing an overview of active ingredients (toxins) include in TSBs, attractants combined in ATSB, lethal effects on mosquito adults and larvae, impact on non-target insects, and prospects for the use of ATSB.

## 2. Toxic Sugar Baits

Modern developments in mosquito control over the last sixty years have focused on sugar baiting for adult mosquito control. Sugar feeding behaviors have been studied for a number of insect vectors such as Culicidae [6,7,8,9], Phlebotominae [10,11,12,13], and Simuliidae [10], demonstrating that many hematophagous dipteran species utilize carbohydrates. Mosquitoes, like other Diptera, may seek and return to carbohydrate sources throughout their life, which presents an opportunity to utilize this behavior for their control [14,15]. Almost all mosquitoes, regardless of larval habitat, require a sugar meal shortly after emergence, and throughout their lives [6,16]. Typically, both male and female mosquitoes have low chances of mating, blood feeding, developing, and laying eggs without energy reserves formed from carbohydrates [6]. For most species of mosquitoes, reserves acquired in the larval stage may only last a few days [6]. Before the larval reserves are depleted, the adult mosquitoes must replenish them by either sugar feeding or blood feeding. To acquire more nutritional reserves mosquitoes will seek out sugars in the environment. Since mosquitoes are liquid feeders, it was theorized that stomach toxins incorporated into a feeding stimulant or arrestant, and placed into environments where mosquitoes were resting, such as larval habitats and foliage near host habitats, might be previously unexplored methods of mosquito control [14].

In 1965, Lea [17] pioneered these sugar-baiting methods in laboratory studies by integrating multiple concentrations (1 mg/mL, 0.5 mg/mL, 0.25 mg/mL, and 0.1 mg/mL) of a toxicant, malathion, into 20% sucrose solutions, formulating the first mosquito toxic sugar bait (TSB) which was fed to *Aedes aegypti* (Linnaeus) on aged (four or 21 days) TSB-treated paper. The mosquitoes readily ate the TSBs with ~85.2% mortality of the adult mosquitoes [17]. From 1990, to the present day, researchers have been following Lea’s TSB methodologies by formulating mosquito baits with low toxicity active ingredients, previously used in baits for other pest species, such as *Bacillus sphaericus* Meyer and Neide, boric acid, and spinosyns [7,18,19]. Sugar baits offer a unique vehicles of pesticide delivery because the sugars trigger an automatic tactical feeding response causing mosquitoes to ingest the integrated active ingredients in the baits and die. Ingested baits may have effects to larval mosquitoes because TSB-affected adult mosquitoes may die over a larval habitat, possibly horizontally transferring toxicants to larval habitats [15]. Due to this uniqueness of the TSB mosquito control method multiple evaluations into the types, and concentrations, of active ingredients that could be incorporated into a TSB started to be explored (Table 1).

Laboratory and field studies of TSBs with sucrose concentrations ranging from 5% to 20%, and multiple active ingredients, have been evaluated for control of Anopheline, Aedine, and Culicine mosquito species [8,14,15,17,19,21,22,23,24,25,26,27]. In laboratory studies using small screened cages, Xue et al. [14] applied a 5% sucrose solution TSB containing 1% boric acid to non-flowering *Rhaphiolepis indica* (L.) Lindl. plants to evaluate the mortality of 100 *Aedes albopictus* Skuse, *Aedes taeniorhynchus* (Wiedemann), and *Culex nigripalpus* Theobald after 48 h exposure to the TSB-covered plants. All mosquito species displayed greater than 96% mortality after exposure to the TSB [14]. During the semi-field portion of Xue et al. [14] study 5000 of each mosquito species were released into large screened cages, one species per cage, with 5% sucrose solution TSB with 1% boric acid applied to multiple plant species in each cage. Xue et al. [14] found that whereas the human landing rate counts (LRC) for *Ae. albopictus* were reduced, the LRC for both *Ae. taeniorhynchus*, and *Cx. nigripalpus* demonstrated little to no reduction [14]. Xue et al. [14] attributed these results, the non-decrease of LRC of *Ae. taeniorhynchus*, and *Cx. nigripalpus*, to the lack of attractive volatiles found in sucrose. The competition of natural sugar sources with attractant components, such as plant volatiles, co-located within the evaluation cages may have caused the mosquito to feed from an alternative plant source and not come into contact with TSBs applied to plants. During field studies, Hossain et al. [27] applied a 5% sucrose solution TSB with 1% boric acid around larval habitat of *Ae. taeniorhynchus*, greatly reducing the landing rate counts of these mosquitoes. TSBs with higher sucrose concentrations of 10%, have primarily focused on studying the mosquito ingestion of multiple active ingredients integrated into TSBs [15,23].

Allan et al. [15] evaluated the susceptibility of active ingredients from five chemical classes: pyrethroids (bifenthrin (7.9%), cyfluthrin (11.8%), deltamethrin (4.75%), permethrin (36.8%), phenylpyroles and fipronil (9.1%)), pyrroles (chlorfenapyr (21.45%)), neonicotinoids (imidacloprid (0.5%), thiamethoxam (21.6%)), and macrocyclic lactones (spinosad (2.46%), ivermectin (0.1%)) in TSB with 10% sucrose solution to *Culex quinquefasciatus* Say, *Anopheles quadrimaculatus* (Say), and *Ae. taeniorhynchus*. This study found that *Cx. quinquefasciatus* was the least susceptible mosquito to many of the active ingredients, for the other two species of mosquitoes the most effective active ingredients were fipronil, delatmethrin, imidacloprid, spinosad, thiamethoxam, bifentrin, permethrin, and cyfluthrin, and the least effective were chlorfenapyr and ivermectrin [15]. Allan’s [15] studies demonstrated that not only can multiple active ingredients be incorporated into TSBs, but they are species-dependent on how well they may work. Further evidence of active ingredients incorporated into TSBs, and their limitations can be seen in studies conducted by Shin et al. [23].

Shin et al. [23], evaluated the individual insecticidal properties of bendiocarb, bifenthrin, cyfluthrin, deltamethrin, etofenprox, permethrin, D-phenothrin, pirimiphos-methyl, α-cypermethrin, and λ-cyhalothrin with a 10% sucrose solution though contact, repellent, and route of action studies (direct contact vs. oral ingestion) on *Culex pipiens molestus* Forskal. The results of these studies determined that these insecticides, when mixed with 10% sucrose solution, behaved more as contact toxicants with <95% mortality through contact, and <15% mortality though ingestion of the insecticides, and that pirimiphos-methyl in sucrose solution was the most repellent TSB to these mosquitoes [23]. Shin et al. [23] studies demonstrate that not all insecticides can be utilized in TSBs for all species of mosquitoes without some possible repellency of the bait.

The studies of TSBs have led to a better understanding of the limitations of these baiting methods. Sugars are not overly attractive and TSB may be outcompeted by natural attractant sugar sources in the environment [14]. The TSBs in the studies mentioned were limited by possible repellency, mosquito species, limited attractiveness, and application placement [8,14,15,17,19,21,22,23,24,25,26,27]. To circumvent these limitations of TSBs, mosquito attractants began to be studied as possible additions to the TSB methods.

## 3. Mosquito Attractants and Attractant Studies

The environment is rich with different attractants that lure mosquitoes to sugar sources, like floral and extra floral nectaries, and rotted fruit or freshly-damaged fruit [6,28,29]. Mosquitoes locate sugar sources through visual attraction, olfactory attraction, and upon tarsal contact of sugars. Once the sugars are contacted, feeding is induced. Flower preference for mosquitoes may be regulated by the circadian rhythm of mosquito behaviors [6,30]. Like other pollinating insects, mosquitoes may be attracted to plants through visual attraction. In studies where night-foraging mosquitoes were collected, most of the mosquitoes were collected from pale-colored or white flowers [6,30]. Some exceptions to light-colored plant attraction have been noted for plants that have strong floral scents, with flower shape not seeming to play a role in mosquito preferences [6,31]. Normal plant sugars are not volatile leading theory that mosquitoes also use olfaction to locate fruits and flowers [31]. Mosquitoes have shown strong attraction, especially when sugar deprived, to flower odors, honey, raw and rotted fruit, and synthetic fragrances in the laboratory [6]. Müller and Schlein [31] found that mosquitoes in arid places were highly attracted to fragrant flowers, which they attributed to the lack of sugar sources and the possibly of a sugar meal being associated with the scent of flowers. Foster [6] indicates that there are components of floral scents that are attractive to mosquitoes: terpenes, phenols, aliphatic esters, and aldehydes. There are limited studies on the effects of combining these floral components for mosquito attraction.

Aside from flowers, fruits produce volatiles that are attractive to sugar-seeking mosquitoes. Theobald [32], in his monograph on Culicidae, describes mosquitoes feeding from banana slices. He further indicates that mosquitoes have been observed walking over fruit and inserting their proboscis into cavitations made by other insects [32]. Other instances of mosquito attraction to fruit come from multiple collections of mosquitoes on damaged and rotted apples, grapes, peaches, and watermelons [33]. Lastly, it has been postulated that mosquitoes may wander around during their resting periods and locate sugars. This is a complex or unknown behavior that has been displayed in other Dipterans while searching for honeydew on leaves [6].

Studies with TSBs were conducted to determine possible attractants that could be used to lure mosquitoes away from their natural sugar sources by offering a “preferred” sugar source. To devise a plant source-attractant for TSBs, fruit, flower, and seedpod attraction studies have been conducted with a number of different mosquito species: *Culex pipiens* L., *Anopheles sergentii* (Theobald), *Aedes caspius* Pallas, *Anopheles gambiae* Giles, and *Ae. albopictus* [7,31,34,35]. The first attractant studies were conducted with locally-available flowering and non-flowering plants [31]. Later, the possible mosquito attractants were broadened by incorporating fruit and edible seeds and insect honeydew as possible attractants [34]. These studies led to the discoveries that male and female mosquitoes are attracted to different sugar sources, and that multiple fruit and seedpods can be used as mosquito attractants [34,35]. The addition of the mosquito host-based attractant, CO_2_, presented with TSB, has been studied with *Ae. aegypti* and *Ae. taeniorhynchus* during semi-field and field studies. TSB (10% sucrose and either 1% boric acid or 0.1% fipronil) was co-located with sachets that produced CO_2_. During the semi field studies, the host-based ATSB was offered to mosquitoes for 48 h resulting in lower landing rate counts than the control cages. The same procedures were used during the field studies, however, the landing rate counts in control and treatment areas were the same, indicating that the addition of host kairomones into baits did not attract and control field populations of *Ae. aegypti* and *Ae. taeniorhynchus* [36].

## 4. Attractive Toxic Sugar Baits Applications for Control of Adult and Larval Mosquitoes

Attractive toxic sugar baits are behavioral manipulation methods that attract adult mosquitoes away from natural sugar sources and induce them to feed from the bait [36,37,38]. When applied to foliage, and in bait stations, these baiting methods capitalize on resting and sugar seeking mosquitoes by being applied where mosquitoes rest, and by attracting them from their natural sugar sources [37,38]. Initial ATSB methods used plants as the attractants. The most attractive plants identified in the study area were sprayed with a color-stained sugar bait, while the same plants in another area were sprayed with TSB [7]. High numbers of mosquitoes (60.5%) captured were observed to have fed from the primitive stained attractive sugar bait (ASB) (non-toxic), with adult mosquito populations feeding from the TSB displaying 91% population control as compared to the ASB control site [7]. The movement away from applying TSB to attractive plants was a result of the effects this methodology poses to non-target organisms, which are also attracted to flowering plants [37]. Jiang and Mulla [39] discovered that in adding a sucrose solution (5%) to form an ingested aqueous bait, the sucrose not only worked as a phagostimulant, but also extended the feeding on the bait and subsequently increased the rate of death in eye gnats (Diptera: Chloropidae). Since many insects are attracted to flowering plants, and application of TSBs to attractive plants may increase the rate of bait consumption, primitive ATSB studies started to capitalize on other plant-based, sugars such as overripe fruit sources that were readily available in the study area, adding sucrose as a phagostimulant, with a toxicant forming rudimentary ATSBs. These primitive ATSBs had an advantage over using flowering plants already in the location as attractants, by being able to dispense fruit-based ATSB on non-flowering plants and around larval habitats to possibly decrease consumption of the baits by non-target organisms.

Multiple attractant fruit and sugar sources have been evaluated in laboratory, semi-field, and field studies that have produced between 36% and 97% mortality to mosquito populations lasting between 8 and 50 days (Table 2). Application methods of ATSBs followed similar patterns as those of TSBs. ATSBs have been applied to foliage in and around larval habitats as spot treatments, in barrier applications [7,9,31,37,40], and in bait stations [8,41,42,43,44,45].

Rudimentary ATSB methods demonstrated varying control of the different adult mosquito species. In an effort to standardized ATSBs to keep consistent mosquito control and demonstrate non-target effects from ATSB applications a, commercial-attractant formulation (Westham Co., Dallas, TX, USA) was developed. The commercial-attractant formulation has been evaluated with multiple active ingredients, such as dinotefuran, eugenol, and garlic oil. Varying levels of mosquito control, 62%–98%, were achieved for Anopheline, Aedine, and Culicine mosquitoes [38,44,48,49] (Table 3). Both rudimentary ATSB and commercially available ATSB methods have been successful in controlling multiple genera of mosquitoes over varying lengths of time. The discrepancies in the percentage of control and duration of treatment observed during rudimentary and commercially available ATSB studies have been attributed to factors of weather [9,46], plant species and flowering state [37,38,40], active ingredient [9,50], and the physiological state of the mosquitoes [43,45,46].

ATSBs and TSBs have focused primarily on adult mosquito control, however, some studies have indicated that there may be larvicidal control though secondary contamination of larval habitats. Schlein and Pener [18] and Schlein and Müller [50] demonstrated that sugar baits have potential to be utilized as vehicles in larval mosquito control. Foliage applications of the sugar plus live *Bacillus sphaericus* (sugar bait) were made on resting sites above *Cx. pipiens* larval habitats, and alongside *An. sergentii* larval habitats. The adult mosquitoes ingested the sugar baits, rested, and died over larval habitat, thus, contaminating the larval habitat with the bacteria [18]. The utilization of sugar as a vehicle to control *Cx. pipiens* and *An. sergentii*, resulted in larval control for up to 22 and ~37 days post application of bait [18,50]. Fulcher et al. [26] demonstrated through simulated rain-wash experiments, that TSBs formulated with insect growth regulator, pyriproxyfen, could control 60%–100% of the larval mosquitoes coming into contact with the wash-off. These studies have provided evidence that ATSB methods may be expanded to include larval mosquito control though secondary contamination of larval habitats.

## 5. Non-Target Insects

An important aspect of IMM practices is to ensure the methods employed in mosquito control are environmentally sensitive [3,4,52,53]. The nature of most pesticides is to block important biological pathways in insects, thusly, insects change their behaviors after coming into contact with pesticides and displaying abnormal behavioral patterns that can lead to inaccurate collections of these insects [38,44,49,51]. To circumvent behavior changes brought about by toxic baits, simulated ATSB applications are conducted through stained ASB applications, to determine the consumption of ATSB by non-target insects.

To date, there have been four field studies on the effects of mosquito sugar-baiting methods on non-target arthropods with all current studies focusing on the non-target effects of the commercially available ASB [38,44,49,51]. To assess the possible primary non-target effects of ATSB applications these studies followed similar baiting and collecting methodologies with commercial attractant formulation-stained and -applied vegetation. Insects fed from the baits for a minimum of 48 h. To ensure an accurate assessment of the insects that fed from ASB, insects where collected with plate traps, pitfall traps, UV traps, sweep nets, and Malaise traps [38,44,49,51].

The major insect orders collected in each study where Coleoptera, Diptera, Hemiptera, Hymenoptera, Lepidoptera, and Orthoptera [38,44,49,51]. These studies determined that the location of the ASB application to flowering or non-flowering plants greatly affects non-target consumption of these baits (Table 4). In all of the studies, Diptera was the most affected order of insect regardless of application to flowering or non-flowering plants [38,44,49,51]. Bait stations provided the best results from non-target studies with most non-targets unable to reach the baits and, as a result, displayed low instances of dyed guts [44].

Secondary exposure to pesticide application can affect predatory arthropods through consuming the insects targeted by the pesticide application [31,44,51]. To evaluate the possible secondary effects of ATSB predators, such as spiders, praying mantises, and predatory coleopterans were fed mosquitoes that had previously fed from, and were engorged with, ATSB [49,51]. None of the predatory arthropods were affected as a secondary result of feeding from the ATSB-engorged mosquitoes [49,51].

ATSB methods have displayed primary and secondary non-target effects with a limited number of non-target arthropod orders studied [49,51]. These studies provide preliminary evidence that ATSB methods have limited effects on non-target arthropods, and allow more arthropod orders to be evaluated through similar bioassay methods.

## 6. Preliminary Studies for Future Applications of ATSB/TSBs

ATSB methods can work as a stand-alone method of mosquito control or in conjunction with other mosquito control methods. Control methods for malarial vectoring mosquitoes rely heavily on indoor residual sprays and long-lasting insecticidal nets that utilize pyrethroids as active ingredients [4,47]. These methods of mosquito control have been incredibly effective, yet their heavy reliance on pyrethroids has resulted in an increase in insecticide resistance [3,4]. Therefore, new techniques of mosquito control, such as ATSB methods, have been suggested to circumvent these problems and prevent malaria resurgence, while continuing to move forward with the elimination of malaria [47].

ATSBs dispersed in bait stations, in conjunction with bed nets, have been evaluated with the idea that host-seeking mosquitoes will deplete their energy reserves trying to access the host. The mosquitoes would then require a sugar meal to regain their energy reserves and imbibe the available ATSB solutions [47]. In field experiments using huts, Stewart et al. [47] evaluated three ATSBs (Table 2) in bait stations against natural populations of mosquitoes (*An. arabiensis*, *Cx. quinquefasciatus*) in conjunction with untreated mosquito nets occupied by human volunteers. Bait stations consisted of paper towels soaked in the respective ATSB, attached to frames and positioned over trays to catch any drippings [47]. The study was conducted to determine if ATSB stations positioned indoors have the potential to kill host-seeking mosquitoes. In the hut trials, mortality rates of the three ATSB treatments ranged from 41% to 48% against *An. arabiensis* and 36%–43% against *Cx. quinquefasciatus* [47]. Stewart et al.’s [47] research has provided examples of how ATSB methods can be utilized to work synergistically with other mosquito control methods.

To circumvent accumulation of pesticide in the environment, and the negative effects of chemical insecticides, other studies have been conducted with Aedine and Anopheline mosquitoes through para-transgenic and transgenic methods by integrating bacteria or double-stranded RNA (dsRNA) into toxic sugar baits [22,24]. In a para-transgenic approach to mosquito control with a sugar bait (SB), Lindh et al. [22] experimented with introducing bacteria (*Bacillus* sp., *Klebisella* sp., *P. stewartii*) into *Ae. aegypti*, and (*P. stewartii*, *Pseudomonas* sp.) into *An. gambiae* s.s. through ingestion of 10% sucrose solution containing sterilized bacterium. Both mosquito species consumed the bacterium SB and the control (10% sucrose solution) in equal quantities [22]. Future studies into para-transgenic mosquito control should focus on SB as vehicles to introduce bacterium into mosquitoes that inhibit pathogens in the guts of mosquitoes [22]. Transgenic approaches to mosquito control through TSBs are beginning to be explored though the introduction of dsRNA into target mosquitos [24]. Coy et al. [24] used mosquito ingestion, and gene knock down to study the effects of introducing dsRNA into mosquitoes through TSB methods. To determine if 3–5 days old *Ae. aegypti* would readily ingest dsRNA, and if the dsRNA was recoverable in the mosquito, the mosquitoes were fed Remebee^®^ (Beeologics, Inc., St. Louis, MO, USA) “a blend of two dsRNA molecules of approximately 480 base pairs RNAi each, which are homologous to the sequence of Israeli Acute Paralysis Virus genome reagent” [24] in varying concentrations (100, 500, 1000, 5000 µL) mixed into 10% sucrose solution, the whole mosquito was used to determine recovery. These experiments deduced that the Remebee^®^ could be recovered and was concentration-dependent [24]. Since the whole mosquito was used to determine if the dsRNA was recoverable, future research into specific locations of the introduced dsRNA in the mosquito body should be conducted. The subsequent studies conducted by Coy et al. [24] involved gene knockdown though feeding *Ae. aegypti* 680 µL Remebee^®^ in 10% sucrose solution for up to 24 h. The mosquitoes were collected at 12, 24, and 48 h, and the RNA was isolated through Ambion’s RNaqueous 4-PCR Kit (Grand Island, NY, USA). Coy et al. [24] found a 2.4–2.5 fold reduction in gene expression for all three of the time periods evaluated. No knockdown mortality was observed during these studies. Lastly, exclusionary bait stations may be necessary for this methodology to exclude non-target insects, and because the dsRNA may be broken down in the environment [24]. These evaluations have determined that certain bacteria and dsRNA can be introduced into mosquitoes using sugar baiting methodologies, and that future research into these methodologies is promising. To date, ATSBs as methods to introduce bacteria or dsRNA have not been evaluated.

## 7. Conclusions

Toxic sugar baiting methods for the control of mosquitoes has been advancing over the last sixty years. The advancements which have been briefly explored in this review include: insect baiting, mosquito sugar baits, mosquito attractants and attractant studies, ATSBs, larvicidal effects of these baits, the effects of ATSB on non-target insects, and future applications of ATSB methodologies. Throughout this review, sugar baiting methods have resulted in the control of multiple mosquito species and low impacts on non-target arthropods. Toxic sugar baits, under certain circumstances, and ATSBs are an effective method of mosquito control that should be continued to advance and be utilized for adult and larval mosquito control, with further research into integrating this method into mosquito abatement programs.

## Figures and Tables

**Table 1:** Active ingredients (AI) and percent AI incorporated into toxic sugar baits and attractive toxic sugar baits for adult mosquito control, by group, subgroup, class, common names, and mode of action (MOA) as indicated by the Insecticide Resistance Action Committee (IRAC) [20].

Group #	Subgroup	Class	Common Name	Mode of Action	% Active Ingredient
1	1A	Carbamates	Bendiocarb	Acetylcholinesterase (AChE) inhibitors	10 g/L
	1B	Organophosphates	Pirimiphos-methyl		
			Malathion		0.10, 0.25, 0.50, 1.0 mg
2	2B	Phenylprazoles (Fiproles)	Fipronil	GABA-gated chloride channel blockers	0.10%
					9.10%
3	3A	Pyrethroids	λ-Cyhalothrin	Sodium channel modulators	10 g/L
			Bifenthrin		
			Cyfluthrin		
			Deltamethrin		
			Etofenprox		
			Permethrin		
			D-Phenothrin (sumithrin)		
			α-Cypermethrin		
			Bifenthrin		7.90%
			Cyfluthrin		11.80%
			Deltamethrin		4.75%
			Permethrin		36.80%
4	4A	Neonicotinoids	dinotefuran	Nicotinic acetylcholine receptor (nAChR) competitive modulators	0.01%
			Imidacloprid		0.50%
			Thiamethoxam		21.60%
5	5	Spinosyns	Spinosad	Nicotinic acetylcholine receptor (nAChR) allosteric modulators	0.04%
					2.46%
6	6	Avermectins, Milbemycins	Ivermectin	Glutamate-gated chlorida channel (GluCl) allosteric modulators	0.10%
7	7C	Pyriproxyfen	Pyriproxyfen	Juvenile hormone mimics	1 mg/L
8	8D	Borates	Boric acid	Miscellaneous non-specific (multi-site) inhibitors	0.0001%, 0.001%, 0.01%, 0.1%, 1%
					0.10%
					0.25%, 0.50%, 0.75%, 1%
					1.50%
					2%
11	11A	Biopesticide	*Bacillus thuringiensis* Berliner.	Microbial gut disruptors	N/A
	11B	Biopesticide	*Bacillus sphaericus*	Microbial gut disruptors	N/A
13	13	Pyrroles	Chlorfenapyr	Uncouplers of oxidative phosporylation via disruption of proton gradient	0.50%
					21.45%
21	21A	METI acaricides and insecticides	Tolfenpyrad	Mitochondrial complex I electron transport inhibitors	1%
N/A	N/A	Double stranded RNA (dsRNA)	Remebee^®^	Endogenous insect gene slicer	0, 100, 500, 1000, 5000 ng/µL
		Botanical	Eugenol	Unknown	0.80%
					1%
			beta-cyclodextrin encapsulated garlic-oil		0.40%
		Biopesticide	*Klebsiella* sp.		1000 bacteria/mL
			*P. stewartii* sp.		
			*Pseudomonas* sp.		
			*P. stewartii* sp.		

**Table 2:** Attractive sugar bait studies (ATSB) by attractant and active ingredient. Data represents attractant sources, percentage of attractants and phagostimulants, references for studies with dates, and results of studies.

Attractant/Phagostimulant	Active Ingredient	Reference # and Year of Study	Mosquito Species	Results/Control
~85% overripe/rotting nectarines and 15% brown sugar	Spinosad	[41] 2008	*An. claviger*	~90%
75% overripe/rotting nectarines and 10% brown sugar	Spinosad	[8] 2008	*Ae. caspius*	91%
			*An. sergentii*	
80% overripe/rotting nectarines and 10% brown sugar	Spinosad	[40] 2010	*Cx. pipens* s.l.	94%
75% overripe/rotting Prickly pear and 20% brown sugar	Boric acid	[37] 2012	*An. sergentii*	~97%
29% Goya Mango juice and 29% Goya Guava juice and 21% brown sugar		[46] 2013	*Ae. albopictus*	~52%
30% overripe/rotting Guava and 30% Honey melon and 12% brown sugar		[45] 2015	*An. gambiae*	~92%
30% overripe/rotting Guava and 30% Honey melon and 12% brown sugar	Boric acid	[9] 2010	*Anopheles arabiensis* Patton	90%
			*An. gambiae*	
95% overripe/rotting Plums and 10% brown sugar	Boric acid	[42] 2010	*Cx. quinquefasciatus*	~85%
95% overripe/rotting Plums and 10% brown sugar	N/A	[43] 2012	*Ae. albopictus*	>90% Stained
			*Anopheles crucians* Wiedemann	
			*Cx. quinquefasciatus*	
			*Toxorhynchites rutilus rutilus* Theobald	
35% Guava juice and 10% brown sugar	Boric acid	[47] 2013	*An. arabiensis*	41%
			*An. gambiae* s.s.	85%
			*Cx. quinquefasciatus*	40%
	Tolfenpyrad		*An. gambiae* s.s.	86%
			*An. arabiensis*	45%
			*Cx. quinquefasciatus*	36%
	Chlorfenapyr		*An. gambiae* s.s.	100%
			*An. arabiensis*	48%
			*Cx. quinquefasciatus*	43%

**Table 3:** Attractive sugar bait studies (ATSB) with commercial-attractant formulation. Data represents attractant sources and phagostimulants, references for studies with dates, and results of studies.

Attractant/Phagostimulant	Active Ingredient	Reference # and Year of Study	Mosquito Species	Results/Control
Commercial-Attractant Formulation	dinotefuran	[51] 2013	*Culex theileri* Theobald	>70%
			*Ae. aegypti*	
			*Ae. caspius*	
			*Culex perexiguus* Theobald	
			*Cx. pipiens*	
			*Cx. quinquefasciatus*	
	Eugenol	[38] 2014	*Aedes infirmatus* Dyar and Knab	94%
			*An. crucians*	62%
			*Cx. nigripalpus*	70%
			*Culiseta melanura* (Coquillett)	55%
			*Uranotaenia sapphirina* (Osten Sacken)	69%
			*Aedes atlanticus* Dyar and Knab	89%
			*Culex erraticus* (Dyar and Knab)	57%
		[44] 2014	*Ae. albopictus*	62%
	beta-cyclodextrin encapsulated garlic-oil	[48] 2015	*Ae. albopictus*	70%
		[49] 2015	*An. sergentii*	81% four days post application, 97.5% overall decline

**Table 4:** Non-target studies conducted with commercially available attractive sugar bait (ASB) with insect orders and families evaluated for consumption effects of bait and secondary effects though consumption-affected mosquitoes.

Reference # and Year of Study	Evaluation Method of ASB	Non-Targets Insect Orders	Percentage of Insects Stained
[51] 2013	Consumption effects-Barrier	Hymenoptera	1.30%
		Lepidoptera	0.60%
		Coleoptera	0.60%
		Diptera	15.00%
		Hemiptera	0.80%
		Orthoptera	1.00%
		Neuroptera	0.30%
[38] 2014	Consumption effects-Barrier	Hymenoptera	15% Flowering, 0.85% Non-flowering
		Lepidoptera	6.71% Flowering, 0.75% Non-flowering
		Coleoptera	5.18% Flowering, 0.69% Non-flowering
		Diptera	17.85% Flowering, 1.45% Non-flowering
		Hemiptera	3.21% Flowering, 0.27% Non-flowering
		Orthoptera	1.25% Flowering, 0.50 Non-flowering
[44] 2014	Consumption effects-Barrier	Hymenoptera	9.2% Flowering, 0.4% Non-flowering
		Lepidoptera	2.5% Flowering, 0.6% Non-flowering
		Coleoptera	3.5% Flowering, 0.5% Non-flowering
		Diptera	11.0% Flowering, 2.1% Non-flowering
		Hemiptera	7.6% Flowering, 0.0% Non-flowering
		Orthoptera	Insect order not evaluated
	Consumption effects-Bait Stations	Hymenoptera	0.003
		Lepidoptera	0.30%
		Coleoptera	0.10%
		Diptera	4.30%
		Hemiptera	Insect order not evaluated
		Orthoptera	0.30%
[49] 2015	Consumption effects	Does not specify	9.2%: 93% of the 9.2% from flowering

## Abbreviations

The following abbreviations are used in this manuscript:

ATSB	Attractive toxic sugar bait
ds-RNA	Double stranded RNA
IMM	Integrated mosquito management
LRC	Landing rate count
MOA	Mode of action
TSB	Toxic sugar bait
WHO	World Health Organization
